# Low overall survival after treatment for angiosarcoma: a single-centre retrospective observational cohort study of 64 patients

**DOI:** 10.1186/s12885-025-14642-7

**Published:** 2025-08-27

**Authors:** Sonja Kjartansdóttir, Erik Malchau, Christina Berger, Emelie Styring, Peter Bergh, David Wennergren

**Affiliations:** 1 Institute of Clinical Sciences, Sahlgrenska Academy, University of Gothenburg, Gothenburg, Sweden; 2 Department of Hand Surgery, Sahlgrenska University Hospital, Gothenburg, Sweden; 3 Department of Orthopedic Surgery, Sahlgrenska University Hospital, Gothenburg, Sweden; 4 Department of Clinical Sciences Lund, Orthopedics, The Faculty of Medicine, Lund University, Lund, Sweden

**Keywords:** Angiosarcoma, Primary angiosarcoma, Secondary angiosarcoma, Epidemiology, Survival

## Abstract

**Background and purpose:**

Angiosarcoma (AS), a rare and highly malignant tumour, can manifest spontaneously (primary AS, pAS) or secondary to previous radiation, exposure to chemical agents or Stewart-Treves syndrome (secondary AS, sAS). The aim of this study was to characterise the clinical presentation, management, treatment and outcome—including local recurrence, metastasis and overall survival—among patients diagnosed with AS and treated at Sahlgrenska University Hospital, Gothenburg, Sweden.

**Study methods and design:**

This is a retrospective single-centre study analysing patients treated for AS at Sahlgrenska University Hospital. Patients were identified over a 21-year period (1 January 2000 to 31 December 2020) using Systematised Nomenclature of Medicine (SNOMED) code M91203. Medical records for all patients were reviewed by the authors.

**Results:**

The study identified 64 patients with AS. The mean age among all patients was 67 (SD 16.6 range 18–96) years. The cohort comprised 37 patients diagnosed with pAS and 27 patients with sAS. Of the patients diagnosed with pAS, 18 (48.6%) were women while 25 (92.6%) women were diagnosed with sAS. Fifty-four (84%) patients were treated with surgery and 34 (63%) with adjuvant chemotherapy, radiation, or both. Local recurrence after primary surgery was 65.4% in pAS and 63% for sAS patients. Upon study completion, 15 (23%) patients were alive. Thirty-nine (60.9%) patients died of AS, while 10 (15.6%) died of other diseases or undetermined causes. Five-year overall survival rates were 10.2% and 43.5% in the pAS and sAS groups, respectively.

**Conclusion:**

pASs are observed in both men and women, presenting at different locations in the body. In contrast, the majority of sASs arise in the thoracic region in women previously treated for breast cancer. The study shows a 60% local recurrence rate across both pAS and sAS groups, yielding 5-year overall survival rates of 10.2% and 43.5% in the pAS and sAS groups, respectively.

## Introduction

Angiosarcoma (AS), a rare and highly malignant neoplasm, arises from mesodermal cells of vascular or lymphatic endothelial origin. It accounts for 1–2% of all sarcomas, representing 0.6% of all cancer disease [1]. Angiosarcoma (AS) most commonly presents as a cutaneous disease in the older people with a male predisposition, but it can arise in bone, soft tissue structure or viscera [2, 3]. Approximately 60% of AS manifestations are cutaneous or in superficial soft tissue, with the head and neck being pre-dominant [4–6]. Most ASs are thought to arise spontaneously, i.e., primary ASs (pASs), but a few predisposing factors, such as previous radiation and chemical agents (vinyl chloride, arsenic), are known. The tumours are classified as secondary AS (sASs) in such cases. Stewart-Treves syndrome constitutes a subgroup of the sASs and develops due to lymphoedema that can be correlated with genetic abnormalities, radiation or previous surgery [1, 7–9].

The management of ASs presents significant challenges because of the non-specificity of symptoms and delayed presentation, often resulting in a diagnosis of advanced or metastatic disease in 16–44% of patients at initial presentation [4–6, 10]. Radical surgery with complete (R0) resection is considered the primary treatment of choice. Achieving an R0 resection in AS is often challenging due to its characteristically multifocal and microscopically invasive growth pattern. R1 and R2 tumour resections are common, with frequent local and metastatic recurrences resulting from residual tumours. Previous studies have shown superior survival when adjuvant radiation is combined with surgery, even with R0 margins [5, 11, 12]. Chemotherapy is recommended as a preferred treatment for patients with metastatic disease, especially with post-operative residual or unresectable tumours [8, 13–15]. Tumour size, type and age have been prognostic factors in some studies [4, 16, 17]. The prognosis is poor, with reported 5-year survival rates of 10–35% [4, 5, 18, 19].

Despite previous studies, there is a lack of contemporary studies on the epidemiology, treatment and outcome of AS. This study aimed to describe the clinical presentation, management, treatment and outcome, specifically addressing local recurrence, metastasis and overall survival of ASs treated at Sahlgrenska University Hospital, Gothenburg, over 21 years.

## Methods

This study is a single-centre retrospective observational cohort study using data from medical records. The patients were identified using the morphological (SNOMED) code M91203 and identified in the records at the Department of Pathology, Sahlgrenska University Hospital. Histopathological diagnoses were made by pathologists with specialised expertise in sarcomas, ensuring a high level of diagnostic accuracy and reliability.

Inclusion criteria were all consecutive patients diagnosed with AS at the Sahlgrenska University Hospital in Gothenburg, from 1 January 2000 to 31 December 2020. Angiosarcoma patients were excluded from the analysis if they were seen only for second opinions without treatment or follow-up.

Data on age, sex, clinical presentation, date of diagnosis, tumour characteristics, metastatic status at diagnosis, and follow-up status were collected by the authors from medical records. The medical records also provided detailed information on tumour grade, size, and histological characteristics.

Before data collection, the study patients were categorised into pAS and sAS groups. Categorisation into these subgroups was generally based on established consensus within the medical records or was explicitly determined during multidisciplinary case conferences. The pAS cohort was defined as patients with no previous history of cancer or radiation in the same localisation, exposure to chemicals or Stewart-Treves syndrome. The sAS cohort was defined as AS related to predisposing factors such as previous cancer or radiation in the same localisation, chemical exposure or Stewart-Treves syndrome.

Descriptive statistics are presented as numbers, percentages and means with corresponding standard deviations. The study defined overall survival as the time from the pathohistological diagnosis until the patient’s death. Kaplan-Meier estimates were used to describe and compare overall survival between groups. Patients surviving to the final follow-up were censored at that point. The log-rank test was used to compare the survival distribution and significance of overall survival between the groups. Statistical significance was established at *p* < 0.05; all analyses were conducted using SPSS version 29.0.2.0 and R.

## Results

**Fig. 1:**
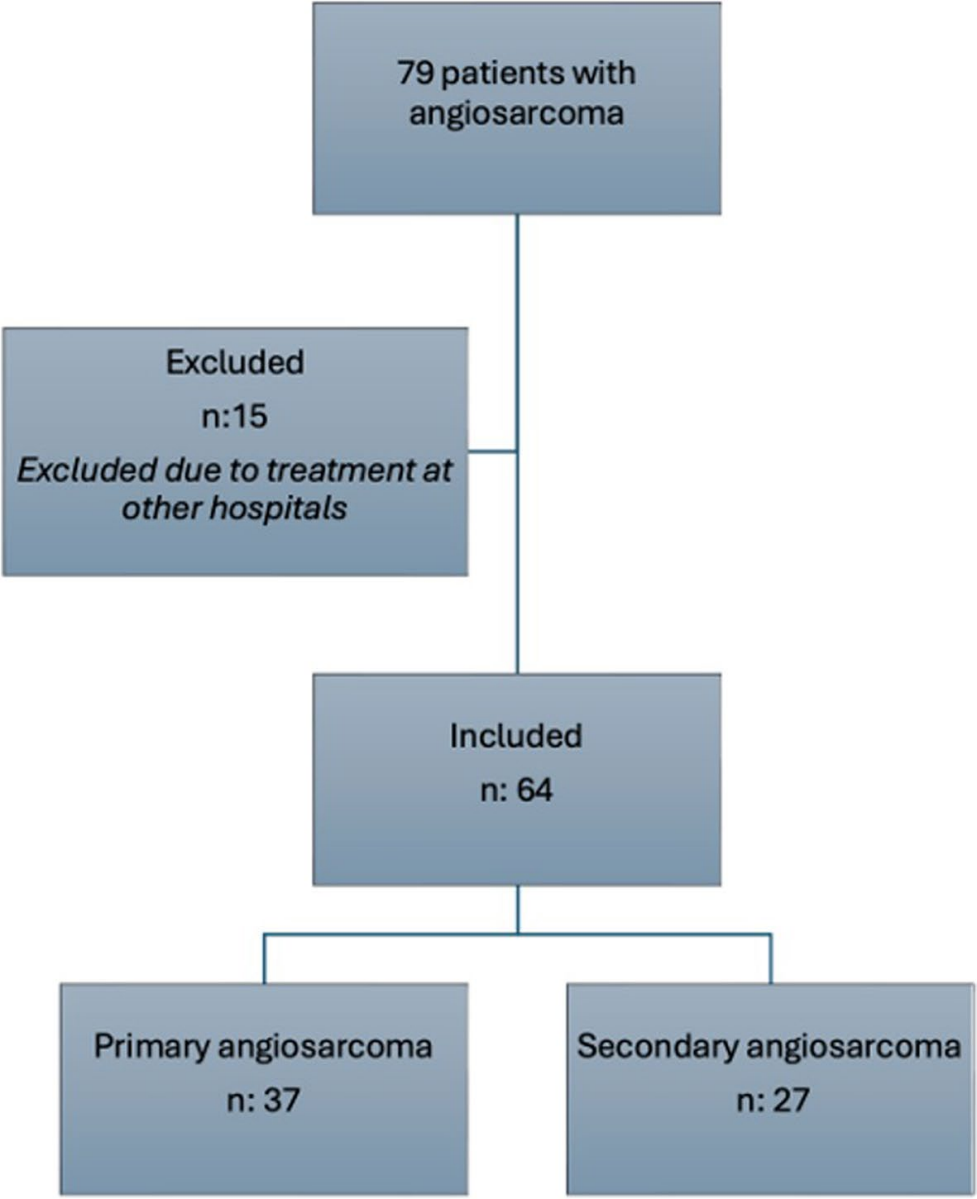
Distribution and division of patients. Fifteen patient tumour samples underwent expert review at Sahlgrenska University Hospital; however, treatment was administered at other hospitals. Thus, these 15 patients were excluded from the study

Overall, 79 patients with a histological diagnosis of AS were identified in medical records at the Department of Pathology, Sahlgrenska University Hospital. Of these 79 patients, 15 (19%) were treated at other hospitals; however, their histological diagnoses underwent review at the Department of Pathology, Sahlgrenska University Hospital for expert consultation and a secondary opinion. These 15 patients were excluded from further analysis. A final cohort of 64 (81%) patients was included in the study (Fig. 1).

Medical records for all patients were reviewed in October 2021, rendering a follow-up time of 1.5–21 years. At diagnosis, all patients underwent CT of the thorax for metastatic staging to evaluate tumour spread.

Women accounted for 43 (67.2%) patients and 21 (32.8%) men. The mean age at diagnosis was 67 (SD 16.6 range 18–96) years. Thirty-seven (57.8%) patients had pASs and 27 (42.2%) sASs. Ten patients had metastatic disease at diagnosis, all of whom presented with pASs. Of the total cohort (*n* = 64), 31 (48.4%) had previous cancer diagnoses. For 6 patients, the first cancer and the AS did not develop in the same area, thus warranting the classification of the ASs as primary.

In the pAS group, 18 (48.6%) were women and 19 (51.4%) men. The mean age at diagnosis was 64 (SD 19.6) years. PASs were most commonly located in the scalp, head and neck (Table 1). The most frequent symptoms at presentation of pASs were pain in the cancer area (*n* = 8, 21.6%) or a palpable lump (*n* = 8, 21.6%). Other common symptoms were a change of skin colour (*n* = 5, 13.5%) or fatigue (*n* = 5, 13.5%). The distribution of symptoms at presentation is presented in Table 1.

**Table 1:** Patient demographics and clinical characteristics. AS, angiosarcoma. pAS, primary angiosarcoma. sAS, secondary angiosarcoma. GI, Gastrointestinal

	Variables	Total AS (*N* = 64) *N* (%)	pAS (*N* = 37) *N* (%)	sAS (*N* = 27) *N* (%)
Mean age at diagnosis (SD)	*Years*	67 SD 16.6	64 SD 19.6	72 SD 9.9
Sex	*Female*	43 (67.2)	18 (48.6)	25 (92.6)
	*Male*	21 (32.8)	19 (51.4)	2 (7.4)
Location of AS	*Breast/thoracic*	24 (37.5)	2 (5.4)	22 (81.5)
	*Scalp, head and neck area*	11 (17.2)	11 (29.7)	
	*Lower extremities*	6 (9.4)	5 (13.5)	1 (3.7)
	*Heart*	5 (7.8)	5 (13.5)	
	*Liver*	4 (6.3)	3 (8.1)	1 (3.7)
	*Upper extremities*	3 (4.7)	1 (2.7)	2 (7.4)
	*Lungs*	3 (4.7)	3 (8.1)	
	*Aorta*,* Vena Cava*	2 (3.1)	2 (5.4)	
	*Spine*	1 (1.6)	1 (2.7)	
	*Upper trunk*	1 (1.6)	1 (2.7)	
	*Thyroid*	1 (1.6)	1 (2.7)	
	*GI duct*	1 (1.6)	1 (2.7)	
	*Spleen*	1 (1.6)	1 (2.7)	
	*Uterus*	1 (1.6)		1 (3.7)
Depth	*Dermal*	9 (14.1)	5 (13.5)	4 (14.8)
	*Subcutanous*	32 (50.0)	11 (29.7)	21 (77.8)
	*Extramuscular (deep)*	4 (6.2)	4 (10.9)	
	*Parenchymal*	19 (29.7)	17 (45.9)	2 (7.4)
Symptom	*Change of skin colour*	17 (26.6)	5 (13.5)	12 (44.4)
	*Lump*	14 (21.9)	8 (21.6)	6 (22.2)
	*Pain in the cancer area*	9 (14.1)	8 (21.6)	1 (3.7)
	*Ulceration of wound*	8 (12.5)	2 (5.4)	6 (22.2)
	*Fatigue*	5 (7.8)	5 (13.5)	
	*Abdominal discomfort*	3 (4.7)	3 (8.1)	
	*Pleural effusion*,* cough*,* hemoptysis*,* dyspnoea*	3 (4.7)	3 (8.1)	
	*No symptom*	2 (3.1)		2 (7.4)
	*Anemia*	1 (1.6)	1 (2.7)	
	*Syncope*	1 (1.6)	1 (2.7)	

Seventeen (45.9%) of the pASs were located in parenchymal organs, including lungs, heart, and liver, whereas 11 (29.7%) were subcutaneous, and 5 (13.5%) were dermal (Table 1).

In a subset of male patients (*n* = 10; 48%), angiosarcoma presented in the scalp and head and neck; the average age at diagnosis was 79 years.

Among the 27 patients with sAS, 25 (92.6%) were women. The mean age at diagnosis was 72 (SD 9.9) years. Initial symptoms most frequently observed were skin discolouration (*n* = 12, 44.4%), ulceration (*n* = 6, 22.2%) and the presence of palpable nodules (*n* = 6, 22.2%). The most common tissue location was subcutaneous (*n* = 21, 77.8%) and dermal (*n* = 4, 14.8%).

In the sAS group, 23 patients (85%) developed angiosarcoma at the same anatomical site as their primary cancer, 22 in the breast/thoracic region and one in the uterus. The breast/thoracic region was the most common location of sAS (*n* = 22, 81.5%). All 22 patients had previously undergone both surgery and radiotherapy for breast cancer. The mean interval between breast cancer treatment and sAS diagnosis was 8.6 years (SD 3.6). In two patients (7.4%), sAS developed in the upper extremity, attributed to prior radiotherapy, mastectomy, or a combination of both. Both had undergone axillary lymph node dissection as part of their initial breast cancer management.

The remaining two patients in the sAS group had no history of cancer but presented with known risk factors for angiosarcoma: one had congenital lymphoedema consistent with Stewart-Treves syndrome, and the other had a history of vinyl chloride exposure.

### Tumour characteristics

Of the tumours examined, 21 showed evidence of necrosis, and 12 demonstrated vascular ingrowth. Infiltrative growth was evident or not determined; no tumours were reported to have pushing growth. It is important to note that many of these parameters remain undetermined (Table 2).

**Table 2:** Analysis of pathohistological reports determining necrosis, vascularity and infiltrative growth. AS, angiosarcoma. pAS, primary angiosarcoma. sAS, secondary angiosarcoma

	All AS (*N* = 64) No (%)	pAS (*N* = 37) No (%)	sAS (*N* = 27) No (%)
Tumour necrosis evident in biopsy/sample	21 (32.8)	12 (32.4)	9 (33.3)
Tumour necrosis not evident in sample	11 (17.2)	7 (18.9)	4 (14.8)
Not determined	28 (43.8)	14 (37.8)	14 (51.9)
Missing	4 (6.2)	4 (10.9)	
Vascular ingrowth evident in sample	12 (18.8)	6 (16.2)	6 (22.2)
Vascular ingrowth not evident in sample	11 (17.2)	5 (13.5)	6 (22.2)
Not determined	37 (57.8)	22 (59.4)	15 (55.6)
Missing	4 (6.2)	4 (10.9)	
Infiltrative growth evident in sample	34 (53.1)	17 (45.9)	17 (63.0)
Infiltrative growth not evident in sample			
Not determined	26 (40.6)	16 (43.2)	10 (37.0)
Missing	4 (6.3)	4 (10.9)	

### Initial treatment and results

Of the total cohort 54 (84.4%) patients with AS underwent surgery. In the pAS group, 27 patients (73.0%) underwent surgery. Of the 10 patients who did not receive surgery, 7 showed evidence of metastatic disease; the remaining 3 patients did not undergo surgery for other reasons. Seventeen patients (65.4%) had local recurrence after primary surgery, of which 9 (52.4%) underwent additional surgery. Thirteen (35.1%) patients with pAS were treated with adjuvant radiotherapy and 22 (59.5%) with chemotherapy.

All patients in the sAS group underwent surgery. Within the sAS group, 17 (63%) patients experienced local recurrence after primary surgery, necessitating further surgical intervention for 12 (71%) of these patients. In the sAS group, adjuvant radiotherapy was administered to 2 patients (7.4%), while 15 (55.6%) received chemotherapy (Table 3).

**Table 3:** Therapeutic measures performed. pAS, primary angiosarcoma. sAS, secondary angiosarcoma

		pAS *n* = 37 No (%)	sAS *n* = 27 No (%)
Operation	*Primary operation*	27 (73.0)	27 (100)
	*Local recurrence 1*	17 (65.4)	17 (63)
	*Reoperation 1*	9 (52.4)	12 (71)
	*Local recurrence 2*	6 (66.6)	10 (83)
	*Reoperation 2*	3 (50)	7 (70)
	*Local recurrence 3*	1 (33.3)	5 (71.4)
	*Reoperation 3*		3 (60)
	*Local recurrence 4*		3 (100)
	*Reoperation 4*		1 (33.3)
	*Local recurrence 5*		1 (100)
Adjuvant radiotherapy	*Yes*	13 (35.1)	2 (7.4)
	*No*	21 (56.8)	25 (92.6)
Chemotherapy	*Yes*	22 (59.5)	15 (55.6)
	*No*	12 (32.4)	12 (44.4)

In the pAS group, 10 (27.0%) patients had metastasis at diagnosis. During follow-up, 20 (54.1%) patients developed metastasis and 7 (18.9%) were metastasis-free at follow-up. All patients in the sAS group were metastasis-free at diagnosis. Twelve (44.4%) patients in the sAS group developed metastasis during follow-up, and 15 (55.6%) were metastasis-free at follow-up (Table 4).

**Table 4:** Metastatic pattern and development of metastasis. pAS, primary angiosarcoma. sAS, secondary angiosarcoma

		pAS *N* = 37 No (%)	sAS *n* = 27 No (%)
Metastasis	*At diagnosis*	10 (27.0)	0
	*During follow up*	20 (54.1)	12 (44.4)
	*Metastasis-free at follow-up*	7 (18.9)	15 (55.6)

At the end of the study 5 (13.5%) of the pAS patients and 6 (22.2%) of the sAS patients survived with active tumours. At the study’s completion, 4 subjects in the sAS group were alive and tumour-free. Thirty-nine (60.9%) patients died of AS, whereas 10 (15,6%) died of other diseases or unknown causes (Table 5).

**Table 5:** Distribution of status at the end of follow up according to primary and secondary angiosarcoma. AS, angiosarcoma. pAS, primary angiosarcoma. sAS, secondary angiosarcoma. NED, no evidence of disease. AWT, alive with tumour. TRD, Tumour-related death. DOC, death of other cause. DUK, death of unknown cause

	NED No (%)	AWT No (%)	TRD No (%)	DOC No (%)	DUK No (%)
pAS (*n*:37)	0	5 (13.5)	25 (67.6)	4 (10.8)	3 (8.1)
sAS (*n*:27)	4 (14.8)	6 (22.2)	14 (51.9)	3 (11.1)	0
Total (*n*:64)	4 (6.3)	11 (17.2)	39 (60.9)	7 (10.9)	3 (4.7)

**Fig. 2:**
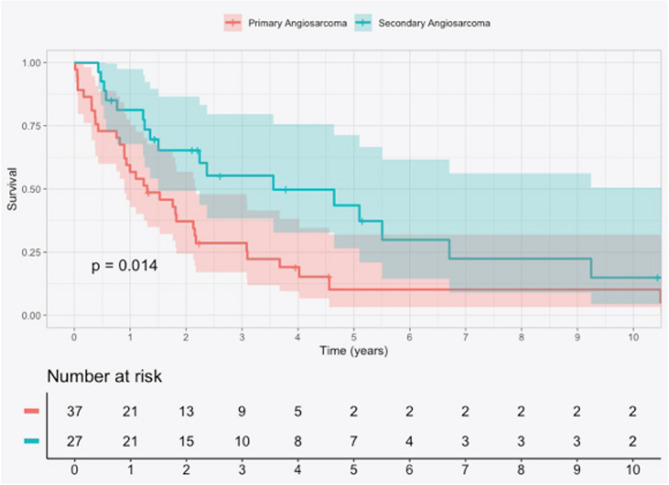
Kaplan–Meier analysis of 10-year overall survival in patients with primary angiosarcoma (pAS) and secondary angiosarcoma (sAS). Shadowed turquoise and pink areas indicate the 95% confidence intervals for each group

The Kaplan-Meier analysis in Fig. 2 depicts the 5-year overall survival of 10.2% (95% confidence interval [CI] 3.2–32%) for pAS and 43.5% (95% CI 26.6–71.4%) for sAS. The median overall survival was 15 months for pAS and 43 months for sAS. A statistically significant difference in survival was seen between the two groups (*p* < 0.05) (Fig. 2).

## Discussion

One of the key findings of this study is the overall poor survival observed among patients treated for angiosarcoma, which underscores the aggressive nature of the disease. Additionally, the analysis revealed a distinct difference in the pattern of metastatic development between primary and secondary angiosarcoma, highlighting potential biological or clinical differences between these subgroups. This study underlines the rarity and aggressive nature of AS, characterised by recurrent episodes and low overall survival despite repeated surgical interventions and/or chemotherapeutic treatments, as previously described [5, 6, 20]. This study shows that, despite treatment for AS, the 5-year overall survival rate is 10.2% for pAS and 43.5% for sAS, with median overall survival times of 15 months and 43 months, respectively. This study’s overall survival results concur with those of Weidema et al. [21].

Histological diagnoses in both our research and the study by Weidema et al. were performed by pathologists experienced in the sarcoma field, ensuring reliable results.

Previous studies have characterised angiosarcoma as a largely asymptomatic neoplasm [12]. Our study revealed that the most common symptoms at initial disease presentation included a small palpable mass, pain in the cancer area, skin discolouration, wound ulceration or fatigue. The presentation of symptoms, often vague, was related to the AS’s location, thus complicating clinical diagnosis. The symptoms, localisation, age and sex reported in this study align with those documented in previous research [6, 7].

One of the biggest challenges in treating AS is the surgical precision for free surgical margins [12, 22]. These patients often require multiple surgical procedures to achieve clear surgical margins, a goal not always attainable. Based on our data, local recurrence rates exceed 60% in patients with pAS and sAS after surgery. In the pAS group, 27 (73%) patients underwent primary surgery and 17 (65.4%) had a local recurrence. All patients with sAS (*n* = 27) underwent surgery and 17 (63%) had a local recurrence. In the pAS group 9 (52%) patients versus 12 (71%) patients in the sAS group required a second operation due to local recurrence. After the second operation, local recurrence exceeded 65% in both groups, necessitating further surgical intervention in some cases.

The elevated local recurrence rate observed in this study may suggest a potential benefit from more extensive surgical resection. Analysis of surgical margins in relation to local recurrence would be of great clinical interest. However, surgical margin assessment in angiosarcoma is difficult due to the diffuse growth pattern of the tumours and its presence in various anatomical sites, where wide excision is often not feasible. These factors lead to inconsistent reporting and limit the ability to evaluate margin reliably. Evaluation of how surgical margins affect local recurrence in one specific location of the body would be an interesting topic for a future study. To conclude, although margin assessment is clearly relevant, it was not the aim of the current study and proved difficult to perform giving the available data.

Styring et al. presented a series of 6 patients with sAS in the breast region treated with excision of the whole radiated field [23]. The absence of metastases at diagnosis in sAS patients suggests that wider surgical margins may improve survival rates [22, 24, 25]. Karlsson et al. showed that the incidence of AS in the breast regions has increased during the past decades [26]. This observation underscores the need to evaluate the effects of excision of the whole radiated field, as proposed by Styring et al.

Notably, the current study revealed that only patients with pAS presented with metastatic disease at diagnosis. At diagnosis 10 (27%) patients with pAS had metastatic disease and 20 (54.1%) developed metastases during follow-up. Only 7 (18.9%) patients were metastasis-free at follow-up.

In contrast to the pAS group, none of the patients with sAS in our study presented with metastatic disease at the time of diagnosis, a finding that, to our knowledge, has not been previously reported. However, given the single-centre design and relatively small sample size, this observation should be interpreted with caution and confirmed in larger, multi-institutional studies. In a study by Hillenbrand et al. with a cohort of 28 patients, distant metastases were observed in 11 patients (61.6%) with pAS and in 4 patients (40.0%) with sAS [21, 27]. Olander et al. showed that the incidence of developing distant metastases was 50% for patients with pAS of the breast versus 31% for sAS of the breast. Nevertheless, the median overall survival was 4.9 years for primary breast AS and 3.6 years for secondary breast AS [9]. While few previous studies have reported somewhat higher rates of metastatic development in pAS, the possibility of a slower or differing metastatic trajectory in sAS has not been thoroughly explored. Our findings raise the question of whether sAS may follow a distinct pattern in metastatic progression, highlighting an area that would benefit from further investigation in larger, prospective studies. These findings may suggest that sAS exhibits a more locally aggressive behaviour with slower metastatic progression, potentially making it more amenable to radical surgical treatment. However, this hypothesis warrants further investigation. In our study during follow up 12 (44.4%) patients with sAS developed metastatic disease and 15 (55.6%) were metastasis-free at follow-up.

Kaplan–Meier analysis demonstrated significantly reduced survival among patients with pAS. This finding aligns with previous reports suggesting a more aggressive disease course in pAS compared to sAS. However, the relatively small sample size in the present study limits the generalisability of these results. Therefore, while the observed trend is noteworthy, it should be interpreted with caution and warrants confirmation in larger, multi-centre cohorts.

Also, in the group of pAS, there were patients with visceral AS who tended to have delayed diagnosis and poorer conditions for radical surgery. Still, the difference in overall survival raises questions about tumour characteristics and behaviour.

Whether pASs exhibit inherently more aggressive behaviour or follow a different clinical timeline remains unclear. It is also possible that a delay in diagnosis compared to sAS contributes to the observed differences. These questions were beyond the scope of the current study, but future research exploring symptom onset, time to diagnosis, and treatment in relation to survival and metastatic progression would be of significant value. In addition, it would be of interest to investigate the role of genetic abnormality, immunotherapy and adjuvant treatments in both sAS and pAS, particularly in relation to their impact on metastatic development and overall survival.

This study has several limitations that should be considered when interpreting the results. Firstly, the retrospective design may introduce information and selection biases, as data was collected from existing medical records, which can be incomplete or inconsistent. Secondly, the study was conducted at a single centre, and all included patients were both diagnosed and treated at the same hospital potentially introducing selection bias and limiting the generalisability of the findings. Third, the rarity of angiosarcoma inherently limits the sample size and the ability to conduct robust subgroup analyses. Additionally, the cohort included angiosarcomas from various anatomical locations, which may differ in biological behavior and clinical outcomes; this heterogeneity limits the ability for site-specific interpretations. Lastly, potential variations in diagnostic and treatment practices over the study period could impact the consistency of care and limit comparability between cases. However, during the study period there were no major changes in clinical practice regarding treatment of angiosarcoma. A strength of the study is that no patients were lost to follow-up. Another strength is that all samples were diagnosed and reviewed by a small group of pathologists at Sahlgrenska University Hospital, all specialised in musculoskeletal tumours.

While our findings are largely consistent with previous studies, we believe this study offers more detailed insights into the epidemiology and clinical characteristics of angiosarcoma, thereby contributing meaningfully to the existing body of knowledge. The ability to compare our results with earlier work highlights both continuity and evolution in the understanding of this rare malignancy. Given the shift toward more conservative surgical approaches and increased use of localised radiotherapy in cancers such as breast cancer, we find it especially important to update epidemiological data and clinical presentations of angiosarcoma. In this context, our up-to-date cohort offers valuable new perspectives.

## Conclusion

pASs affect both men and women, manifesting in different locations in the body; however, most sASs in women with a history of breast cancer arise in the thoracic region. This study shows a high incidence of local recurrence and low overall survival after treatment for both primary and secondary AS. We found a statistically significant difference in overall survival for primary and secondary AS, with a 5-year overall survival of 10.2% in the pAS group and 43.5% in the sAS group. Understanding the epidemiological characteristics and biological behaviour of angiosarcoma provides a foundation for future research into improved treatment.

## Data Availability

The data supporting this study’s findings are available from the corresponding author upon reasonable request. Data are in controlled access data storage at Sahlgrenska University Hospital.

## Abbreviations

AS: Angiosarcoma
pAS: Primary angiosarcoma
sAS: Secondary angiosarcoma
NED: No evidence of disease
AWT: Alive with tumour
TRD: Tumour-related death
DOC: Death of other cause
DUK: Death of unknown cause
OS: Overall Survival
GI: Gastrointestinal
